# Altered Feeding Behaviors and Adiposity Precede Observable Weight Gain in Young Rats Submitted to a Short-Term High-Fat Diet

**DOI:** 10.1155/2018/1498150

**Published:** 2018-04-01

**Authors:** David E. Andrich, Lilya Melbouci, Ya Ou, Jean-Philippe Leduc-Gaudet, François Chabot, François Lalonde, Fábio Santos Lira, Bruce D. Gaylinn, Gilles Gouspillou, Gawiyou Danialou, Alain-Steve Comtois, David H. St-Pierre

**Affiliations:** ^1^Département des Sciences de l'Activité Physique, Université du Québec à Montréal (UQAM), 141 President-Kennedy Ave., Montréal, QC, Canada H2X 1Y4; ^2^Groupe de Recherche en Activité Physique Adaptée (GRAPA), UQAM, 141 President-Kennedy Ave., Montréal, QC, Canada H2X 1Y4; ^3^Département des Sciences Biologiques, UQAM, 141 President-Kennedy Ave., Montréal, QC, Canada H2X 1Y4; ^4^Centre de Recherche du CHU Sainte-Justine, 3175 chemin de la Côte-Sainte-Catherine, Montréal, QC, Canada H3T 1C5; ^5^Department of Physical Education, São Paulo State University, 19060-900 Presidente Prudente, SP, Brazil; ^6^Department of Medicine, University of Virginia, 450 Ray C. Hunt Drive, Charlottesville, VA 22903, USA; ^7^Royal Canadian Military College, 15 Jacques-Cartier Nord, Saint-Jean-sur-Richelieu, QC, Canada J3B 8R8

## Abstract

Information regarding the early effects of obesogenic diets on feeding patterns and behaviors is limited. To improve knowledge regarding the etiology of obesity, young male Wistar rats were submitted to high-fat (HFD) or regular chow diets (RCDs) for 14 days. Various metabolic parameters were continuously measured using metabolic chambers. Total weight gain was similar between groups, but heavier visceral fat depots and reduced weight of livers were found in HFD rats. Total calorie intake was increased while individual feeding bouts were shorter and of higher calorie intake in response to HFD. Ambulatory activity and sleep duration were decreased in HFD rats during passive and active phase, respectively. Acylated and unacylated ghrelin levels were unaltered by the increased calorie intake and the early changes in body composition. This indicates that at this early stage, the orexigenic signal did not adapt to the high-calorie content of HFD. We hereby demonstrate that, although total weight gain is not affected, a short-term obesogenic diet alters body composition, feeding patterns, satiation, ambulatory activity profiles, and behaviours in a young rat model. Moreover, this effect precedes changes in weight gain, obesity, and ensuing metabolic disorders.

## 1. Introduction

Obesity and ensuing metabolic dysfunctions are major issues for public health authorities [1–3]. This is of particular concern in pediatric populations in Canada where excess weight and obesity have reached the prevalence of 20% and 13%, respectively [4]. Early adoption of detrimental behaviors such as overeating, consumption of energy-dense foods, and lack of physical activity promote excessive weight gain. This is likely to have a major impact in later life stages since 85% of obese children become obese adults [5, 6]. Further, the initial mechanisms promoting obesity and ensuing metabolic complications remain to be better characterized by using dynamic experimental designs rather than classical static protocols. This highlights the relevance of using animal models to characterize the early steps leading to obesity and its evolution towards further pathological conditions. Obesogenic diets greatly contribute to a positive energy balance and their fat content provides approximately twice the caloric load of carbohydrates and proteins [7]. Beyond the simple energy intake/expenditure equation, high-fat diets (HFD) are suspected to promote the development of obesity through other indirect mechanisms. For instance, prior to weight gain, a significant increase in total triglyceride levels was reported after only 2 weeks in female rats submitted to HFD [8, 9]. This suggests that important physiological changes can occur early in response to HFD, and this can be detected before significant increases in body weight.

Early effects of HFD include changes in critical behaviors such as feeding, physical activity, and sleep. For instance, rats submitted to HFD for 8 weeks decreased their physical activity levels by 28% [10]. In mice, obesogenic diets were also shown to alter feeding behaviors and voluntary activity [11]. Independently of body weight fluctuations, these observations imply potential impairments in the regulation of vital functions such as appetite, satiation, satiety, energy utilization/storage, sleep, and voluntary activity [12]. It is reported that only one week of HFD is sufficient to alter eating behaviors in adult mice [13]. Although these alterations are quickly reversed after animals are resubmitted to a standard diet [14], the early effects of HFD could negatively influence lipid and glucose homeostasis while triggering the onset of obesity-related disorders in the long term. Similarly to what is reported in mice, young rats are particularly sensitive to the dysregulation of some vital physiological functions in response to HFD [15]. This emphasizes the important adverse repercussions of an obesogenic diet in early life stages.

Critical changes in behaviors were previously noted in rats submitted to HFD for different time periods. For instance, heat production was increased before changes in weight gain, and energy intake could be detected in young rats submitted to HFD for 1 and 2 weeks [10]. Shorter feeding bouts of higher calorie content were observed before changes in weight gain in young adult rats submitted to 5 weeks of HFD [16]. However, it is currently unknown whether these changes in feeding behaviors occur earlier in response to HFD. Decreased ambulatory activity was observed after 8 weeks of HFD in adult rats [10]. Further, altered sleep patterns [17] and alternating periods of food accessibility [18] are proposed to contribute to weight gain and abdominal adiposity in rats. However, there is a critical gap of knowledge regarding how ambulatory activity and sleep are modulated by obesogenic diets over time. A lack of adaptation in the secretion of neuroendocrine orexigenic signals to calorie-dense diets could explain these phenomena. In physiological conditions, ghrelin levels raise in anticipation of a meal and decrease during the postprandial period [19]. However, postprandial acylated ghrelin (AG) levels also augment in response to stress, overfeeding, and high-fructose or high-fat diets in animals or humans [20–23]. This could explain why satiation and satiety do not adapt in response to energy-dense diets.

Most of the useful information on the etiology of obesity is derived from experiments achieved on defined set points in adult rats. However, the present study intended to characterize changes in behaviors with a dynamic model of analysis through continuous noninvasive monitoring in metabolic chambers. Such experimental protocols were critical in investigating the synergy between diets, feeding and behaviors as well as to determine their repercussions on the early steps leading to obesity in young rats.

## 2. Materials and Methods

### 2.1. Animal Procedures

Young (100–125 g; approximately 4 weeks old) male Wistar rats (Charles River, St-Constant, QC) were randomly submitted to HFD (*n*=8) or regular chow (RCD; *n*=8) diets, *ad libitum*, after a 3-day acclimatization period at UQAM's animal facility. During the experimental procedures, rats were individually housed in metabolic chambers (at 22 ± 2°C) in Oxymax CLAMS (Columbus Instruments, OH, USA) for 14 days and submitted to a 12-hour light/dark cycle starting at 06:00. The Oxymax CLAMS system allowed measuring food/water intakes, feeding patterns, energy expenditure, ambulatory activity, and sleep profiles. Also, rats were individually weighed daily at the same hour. Sacrifice was achieved after a 4-hour fast to standardize the feeding status of each animal. Blood samples were then drawn from the heart. Perirenal, epididymal, and subcutaneous fat pads were then collected and weighed. This study was carried out in strict accordance with recommendations of the National Institutes of Health guide for the care and use of Laboratory animals. The protocol was approved by the Comité Institutionnel de Protection des Animaux (CIPA) of UQAM (Permit Number: 0515-R3-759-0516). All efforts were made to minimize any potential discomfort to the animals.

### 2.2. Diets

The regular chow diet (Charles River Rodent Diet #5075, Cargill Animal Nutrition, MN, USA) had a physiological fuel value (calculated using a modified Atwater factor) of 2.89 kcal/g and a macronutrient weight content of 55.2% carbohydrate (65.6% kcal), 18% protein (21.4% kcal), and 4.5% fat (13% kcal). The high-fat diet was prepared from purified food-grade reagents according to a commercial formulation (D12492 diet, Research Diets Inc., New Brunswick, NJ, USA). It had a physiological fuel value of 4.80 kcal/g and a macronutrient weight content of 26.3% carbohydrate (19.2% kcal), 26.2% protein (19% kcal) and 34.9% fat (61.8% kcal). Protein sources were casein and L-cystine (98.5% and 1.5%, resp.) and lipid sources were lard and soybean oil (90.7% and 9.3%, resp.) while carbohydrate sources were maltodextrin and sucrose (64.5% and 35.5%, resp.). The diet also contained cellulose (64.6 g/kg), calcium carbonate (7.1 g/kg), dicalcium phosphate (16.8 g/kg), potassium citrate (21.3 g/kg), and choline bitartrate (2.6 g/kg) as well as mineral (12.9 g/kg) and vitamin (12.9 g/kg) mixes (Table 1).

### 2.3. Metabolic Chambers

The CLAMS system's O_2_ and CO_2_ sensors were calibrated every second day in accordance with the manufacturer's instructions, as previously described [24]. Measurements of O_2_ and CO_2_ (mL/kg/hr) were achieved using open-circuit indirect calorimetry system by calculating the difference between [O_2_] consumption and [CO_2_] production. Furthermore, the respiratory exchange ratio (RER), highlighting the relative contributions of lipid and glucose oxidation, was calculated from the aforementioned volumetric values ([CO_2_]/[O_2_]). Energy expenditure (kcal/hr) was estimated by the Oxymax CLAMS software using the following equation: energy expenditure *=* CV ∗ [VO_2_], where CV (i.e., calorie value) = (3.815 + 1.232 ∗ RER) [25, 26]. For each metabolic chamber, the Oxymax CLAMS system used an individual balance (PL1502E, Mettler Toledo, Switzerland) allowing accurate in situ monitoring (±0.01 g precision) of food intake and feeding bouts (weight and duration). Feeding bouts were computed when the feeder's balance was used for more than 10 s or more than 0.1 g of food was ingested. Also, the system's unique design optimally minimized food spillage and foraging. Volumetric drinking (mL) was calculated using the sipper tube technology. Energy balance (energy balance = daily calorie intake − daily energy expenditure) was also calculated from aforementioned parameters [27, 28]. Ambulatory activity was continuously assessed using infrared photocells on the *X* and *Z* axis. It was calculated when the animal broke a series of infrared beams in sequence, thus excluding stereotypy. Sleeping time was computed when animals did not break a single infrared beam for more than 60 s, as previously described [29, 30]. All parameters were collected and analyzed using the Oxymax CLAMS software and continuously monitored throughout the 14 days of the experimental protocol.

### 2.4. Ghrelin Assay

Collected blood was added to chilled EDTA Vacutainer tubes preloaded with 4-[2-aminoethyl benzene] sulfonylfluoride (Alexis Biochemicals), then stored on ice. Within 1 hour of collection, blood was centrifuged for 10 minutes at 2000 ×g at 4°C. After separation, 0.5 mL plasma was acidified with 100 *μ*L of 1 N HCl. Samples were then stored at −20°C. Plasma acylated and unacylated ghrelin concentrations were measured with a two-site sandwich ELISAs, as previously described [31]. Briefly, plates (384-well Maxisorb; Nunc, Roskilde, Denmark) were coated with acyl-specific antiserum (acylated) or affinity-purified C-terminal ghrelin antiserum (unacylated) at 1 *μ*g/mL overnight. After being blocked, the plate was washed and loaded with 25 *μ*L/well wetting/neutralization buffer (0.5 M phosphate buffer with 1% BSA, pH 7.4) and 25 *μ*L/well ghrelin standards or unknown samples and incubated overnight at 4°C. The washed plate was incubated for 1 h with either a biotinylated C-terminal ghrelin antiserum (acylated) or a biotinylated N-terminal des-acyl ghrelin-specific monoclonal antiserum (unacylated) in blocking buffer, then for 30 min with streptavidin-poly-HRP80 (RDI Fitzgerald, Concord, MA). The plate was detected with the fluorescent substrate QuantaBlu (Pierce Thermo, Rockford, IL), and fluorescence was read using a Victor2 Model 1420 (PerkinElmer, Waltham MA). All unknowns were run in duplicate, and all samples for each admission of each rat were run on the same plate. Standards were made up in acid/AEBSF-treated stripped plasma. Percentage of acylation was calculated using the following formula: 100 ∗ AG/(AG + UAG).

### 2.5. Statistical Analyses

All values are presented as means ± SD. Distribution normality was assessed using the Shapiro-Wilk test. Unpaired Student's *t*-tests were used to compare 14-day average values between the two groups. Mixed linear regression models were used to compare the evolution of various parameters over 14 days between the two groups. Each distribution was tested for linearity beforehand. Statistical analyses were performed using the SPSS 16.0 (IBM Corporation, Armonk, NY) and SAS Studio 3.5 (SAS Institute, Cary, NC) softwares. Significance for all statistical analyses was set at *P* < 0.05.

## 3. Results

**Figure 1:**
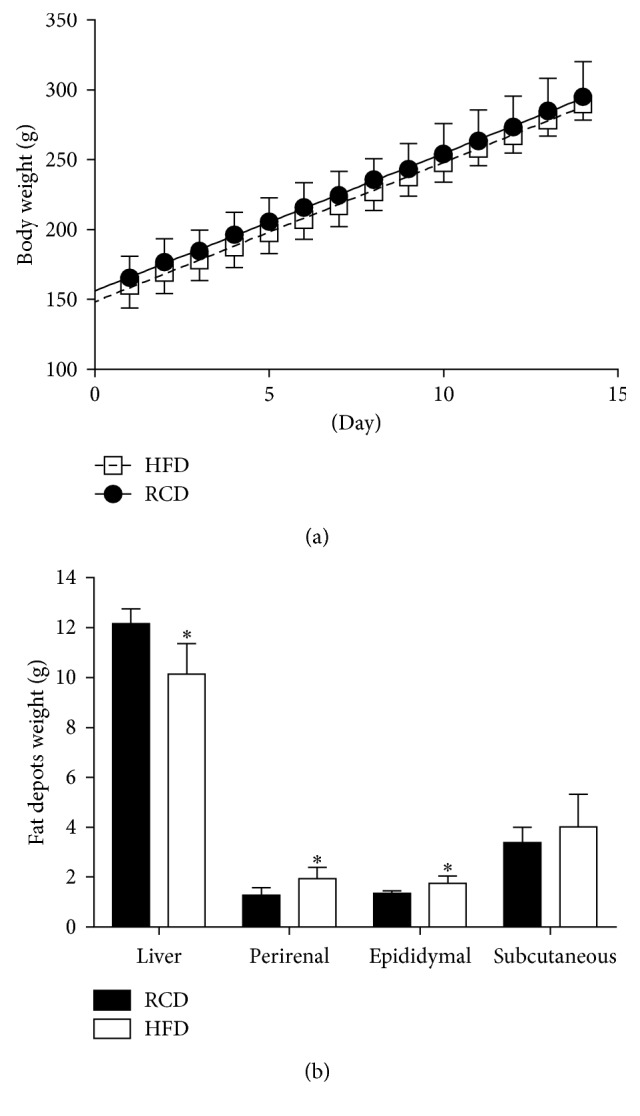
Body weight evolution (a) and average weights for livers, perirenal, epididymal, and subcutaneous abdominal fat pads (b) in young rats submitted to HFD or RCD for 14 days. Results are presented as means ± SD; ^∗^ indicates significant difference between the two diets (*P* < 0.05) using an unpaired Student's *t*-test.

After submitting the animals to the different diets for 14 days, body weight gain (Figure 1(a)) was similar in both groups and followed the same trend. However, significant differences in organs and tissues were observed at the end of the 14-day period. Average liver weight was lower (*P*=0.001) while those of visceral perirenal (*P*=0.003) and epididymal (*P*=0.004) fat pads were higher in HFD than in RCD rats (Figure 1(b)). On the other hand, the weight of subcutaneous fat pads was similar in both groups.

**Figure 2:**
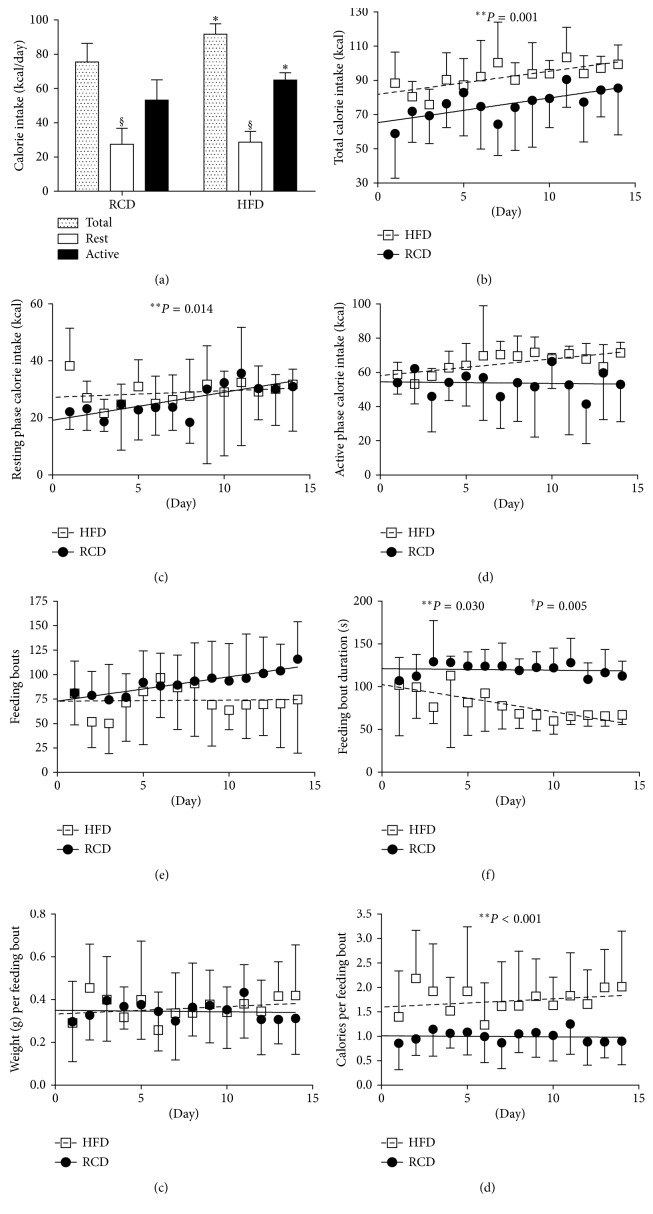
Total (a-b), resting phase (c), and active phase (d) calorie intake; number of daily feeding bouts (e), average feeding bout duration (f), and weight (g) as well as calories per feeding bout (h) in young rats submitted to HFD or RCD for 14 days. Results are presented as means ± SD; ^∗^ indicates significant difference between the two groups (*P* < 0.05) and § indicates significant difference from nocturnal value at the *P* < 0.05 level using an unpaired Student's *t*-test; ^∗∗^ indicates significant difference between the two groups (*P* < 0.05) and † indicates a significant group × time effect (*P* < 0.05) using a mixed linear regression test.

Average daily water and macronutrient intakes are shown in Table 2. Total protein and water intakes were similar between both groups (*P*=0.771 and *P*=0.091, resp.). Further, ingested carbohydrates (g/day; *P* < 0.001) were significantly higher in the RCD group. As expected, fat intake (g/day) was more important in the HFD group (*P* < 0.001). Calorie intakes (Figures 2(a)–2(d)) were significantly higher in the HFD group over the total experimental protocol (*P*=0.001) and the resting (light) phase (*P*=0.014) while no difference was detected during the active (dark) phase (*P*=0.381). The number of feeding bouts was similar between HFD and RCD rats (*P*=0.926; Figure 2(e)). However, feeding bout duration was lower in HFD than that in RCD rats (*P*=0.030; Figure 2(f)), and a significant group × time effect (*P*=0.005) was also detected. It is also noteworthy that the weight of individual feeding bouts was similar in both groups (*P*=0.663; Figure 2(g)); thus, HFD rats ingested significantly more calories per bout than RCD rats (*P* < 0.001; Figure 2(h)), suggesting the alteration of mechanisms regulating satiation.

**Figure 3:**
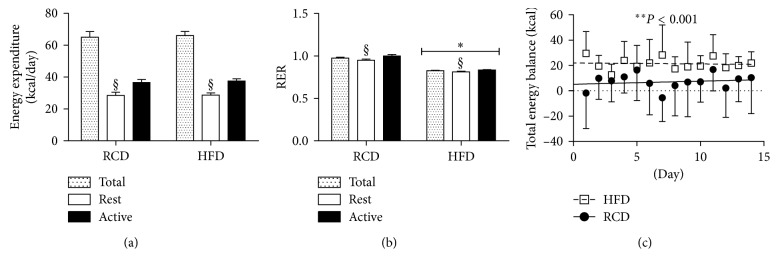
Energy expenditure (a) respiratory exchange ratio (RER) (b) and total energy balance (c) values in young rats submitted to HFD or RCD for 14 days. Results are presented as means ± SD; ^∗^ indicates significant difference between the two groups (*P* < 0.05) and § indicates significant difference from nocturnal value at the *P* < 0.05 level using an unpaired Student's *t*-test.; ^∗∗^ indicates significant difference between the two groups (*P* < 0.05) using a mixed linear regression test.

Average energy expenditure was equivalent in both groups (HFD: 70.4 ± 2.8 kcal/day versus RCD: 69.4 ± 3.8 kcal/day; *P*=0.5; Figure 3(a)). Further, energy expenditure was significantly higher during the active phase for both groups. As expected, HFD rats displayed lower respiratory exchange ratio (RER) values (HFD: 0.826 ± 0.006 versus RCD: 0.975 ± 0.012; *P* < 0.001; Figure 3(b)). Higher calorie intakes and similar energy expenditure resulted in a more positive energy balance in the HFD group (*P* < 0.001; Figure 3(c)).

**Figure 4:**
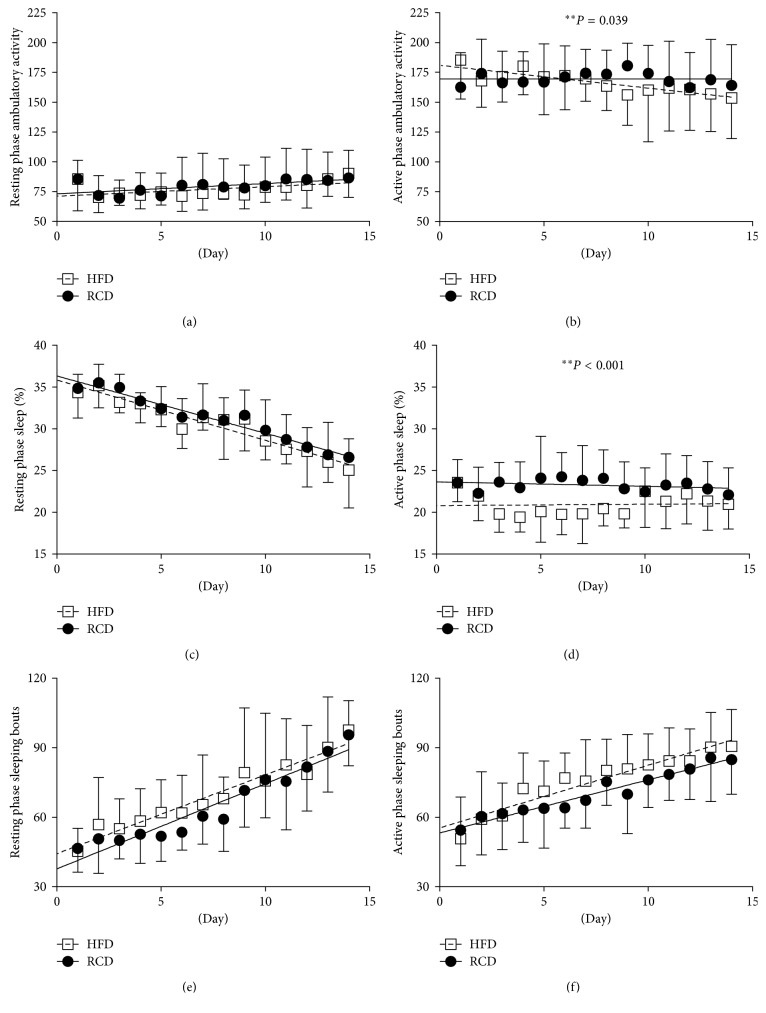
Resting phase (a) and active phase (b) ambulatory activity; resting phase (c) and active phase (d) percent sleeping time; resting phase (e) and active phase (f) sleeping bouts in young rats submitted to HFD or RCD for 14 days. Results are presented as means ± SD; ∗∗ indicates significant difference between the two groups (*P* < 0.05) using a mixed linear regression test.

There was a significant difference in ambulatory activity between HFD and RCD rats during the active phase (*P*=0.039; Figure 4(b)). However, this effect was not observed during the resting phase (*P*=0.582; Figure 4(a)). Interestingly, over the progression of the experimental procedures, ambulatory activity associated to the active phase decreased in HFD while remaining stable in RCD rats. Percent sleeping time was significantly lower in the HFD group (*P* < 0.001; Figure 4(d)) during the active phase, but this effect was not observed over the resting period (*P*=0.496; Figure 4(c)). No difference in the number of sleeping bouts was detected during the resting (*P*=0.069; Figure 4(e)) or the active (*P*=0.454; Figure 4(f)) phases.

**Figure 5:**
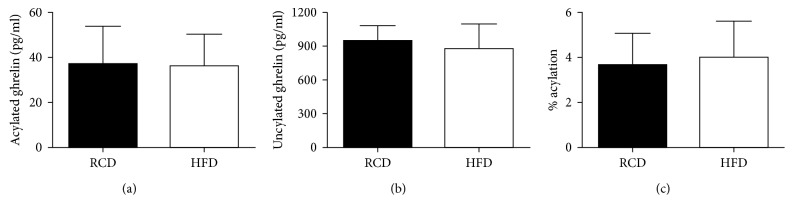
Acylated ghrelin (a) and unacylated ghrelin (b) plasma concentrations as well as percentage of acylation (c) in young rats submitted to HFD or RCD for 14 days. Results are presented as means ± SD.

Acylated ghrelin plasma concentrations were similar in both groups (HFD: 36.07 ± 14.13 versus RCD: 36.71 ± 16.95 pg/mL; *P*=0.94; Figure 5(a)). Further, no significant difference could be observed between both groups in unacylated ghrelin levels (HFD: 878.30 ± 215.93 versus RCD: 946.34 ± 133.32 pg/mL; *P*=0.49; Figure 5(b)) or percentage of acylation (HFD: 4.01 ± 1.59 versus RCD: 3.67 ± 1.40%; *P*=0.68; Figure 5(c)).

## 4. Discussion

In the present study, the use of metabolic chambers was critical to characterize how young rats respond to HFD. To our knowledge, this is the first study that simultaneously assessed metabolic and behavioral parameters continuously in a noninvasive manner over a 14-day period in young animals. Major findings of this study are that reduced satiation, active phase ambulatory activity, and percent sleep time occur before changes in weight gain in young rats submitted to HFD for a period of only 14 days. This indicates that important changes in feeding and behaviors arise early in the process leading to the excessive accumulation of fat in young growing rats. In turn, if not promptly reverted, this could promote the development of obesity and ensuing metabolic dysfunctions in later life stages.

Similar overall weight gain was observed in HFD and RCD groups over the 14-day experimental protocol. This finding is significant since short-term exposure to HFD also yielded increases in calorie intakes and impairments in the regulation of satiation. Further, these results were associated with reduced liver weight and increased mass of visceral fat pads in animals fed with this obesogenic diet. This indicates that although young rats submitted to HFD were still able to counteract excessive weight gains, alterations in body composition occurred while the mass of visceral fat depots simultaneously increased. The present results are in line with the equivalent weight gain observed over a period of up to 3 weeks in Wistar rats submitted to HFD or RCD [10]. Similar weight gains were also reported for the first 4 weeks of HFD in young Sprague Dawley rats [16]. In contrast to what was observed in rat studies, only one week of HFD was sufficient to promote significant changes in weight gain and insulin resistance in aged C57BL6 female mice [32]. Also, reductions in liver weight combined to an excessive accumulation of fat in hepatic tissues were reported in the offspring of female rats submitted to HFD during pregnancy and lactation [33]. Further, the lower liver weight observed in response to the short-term HFD could be a consequence of lower glycogen storage [34] or other uncharacterized mechanisms. Data derived from the present study strongly indicate the importance of determining the effects of HFD before weight gain occurs in young rats. Further, these results shed the light on feeding patterns and behaviors that are influenced promptly by HFD and that could have a major impact on the early alterations promoting the development of obesity in younger populations.

In addition to the metabolic adaptations observed in response to an obesogenic diet, the present results show that eating habits are altered by the short-term exposure to HFD. Impairments in the regulation of timing and rhythmicity of feeding profiles are associated to the development of obesity in rodents and humans [35]. In the present study, the number of feeding bouts was the same between young rats submitted to HFD or RCD. Over time, however, the duration of feeding bouts was reduced while calorie intakes became higher in HFD rats. This contradicts the previous assertion that the development of obesity results from a reduction in meal numbers and an increase in snacking events in rats submitted to a highly palatable cafeteria diet [36]. However, data derived from the present study show that HFD promotes the ingestion of excessive amounts of calories before satiation can be reached. It is critical to consider that afferent endocrine signals require approximately 20 minutes to be secreted and integrated after feeding [37]. As previously reported, the regulation of appetite and satiation is a complex process involving a plethora of neuroendocrine signals derived from key organs and tissues such as the gastrointestinal tract and adipose tissues. These signals are integrated by specific centers of the brain. In the present study, no difference in AG or unacylated (UAG) ghrelin levels were detected. This result is of particular interest when considering that AG induces a potent orexigenic signal that acts primarily through its activation of neuropeptide Y (NPY) and agouti-related protein (AgRP) neurons of the arcuate nucleus that are projecting to other centers responsible for the physiological control of food intake in the hypothalamus [38–40]. Ghrelin was also reported to stimulate appetite by activating hedonic centers in the brain [41]. In physiological conditions, AG levels are increased in preprandial condition and decrease after the consumption of a meal [19]. However, postprandial AG levels were also shown to increase in response to stress, overfeeding, and high-fructose or high-fat diets in animals or humans [20–23,42,43]. This suggests that AG's orexigenic signal could be perpetuated when other neuroendocrine satiation hormones should prevail, thus leading to exaggerated calorie intakes. On the long run, ghrelin's adipogenic effects [19, 44] could also promote the excessive accumulation of lipids in different adipose tissues and liver [45, 46]. This could ultimately provoke the onset of obesity-related diseases.

In this study, energy expenditure was found to be similar in both groups regardless of the diet. Our results, obtained in response to a 14-day treatment period, are in line with those of Furnes et al., indicating that no alteration of energy expenditure occurs in young Sprague Dawley rats submitted to a HFD or a RCD for 5, 17, and 33 weeks [16]. In contrast, energy expenditure was significantly increased during the initial 2-week period in adult Wistar rats submitted to HFD [10]. However, this increase was transient and did not persist after the second week. As expected, lower RER values were observed in HFD rats when compared to the RCD group. This confirms the prompt metabolic shift towards lipid oxidation that takes place in young rats submitted to HFD [10].

The present results show that the ambulatory activity decreases with time during the active phase in HFD rats while remaining stable in RCD rats. This is in line with data obtained after 8 weeks of HFD in adult rats [10]. It is noteworthy that voluntary physical activity was previously shown to counteract the effects of hyperphagia and of an obesogenic diet in young and aged rats [12, 47]. In human adults prone to obesity, it was suggested that increased spontaneous physical activity helps preventing weight gain induced by overfeeding [48]. This is also supported by the increased physical activity observed in normal-weight young adults submitted to overfeeding for 56 days [49]. In addition, it was proposed that spontaneous physical activity is a major predictor of weight gain in overfed humans [50]. This further supports the hypothesis that over time, obesogenic diets increase weight gain by promoting both high calorie intakes and sedentary behaviors. Finally, it has also been shown that sleep deprivation can promote obesity in rodent models and humans [51]. Lower percent sleeping time was measured throughout the experiments using HFD in our model, and the mechanisms underlying this effect remain to be elucidated. Further, a steady decline in sleep quality was observed in both groups during the 14-day period.

## 5. Conclusions

In conclusion, the present results show the pertinence of measuring key metabolic parameters and behaviors in response to obesogenic diets in a young growing rat model on a continuous basis. This study demonstrates that at a very early stage, HFD alters key functions related to appetite, feeding behaviors, satiation, fat storage, physical activity, metabolism, and sleep. It suggests that energy-dense HFD could induce a vicious cycle that promotes higher calorie intake and sedentary behaviors, two major risk factors for the development of obesity and ensuing metabolic dysfunctions. Further, the results presented herein imply the existence of a transitory state where young animals accumulate visceral adiposity while increases in weight gain are not yet detectable in response to hypercaloric intakes. However, this transitory state may only last between 2 and 4 weeks and could develop further into obesity. We anticipate that the results derived from the present study could be applied to elaborate novel interventions in nutrition and physical activity to circumvent obesity and ensuing metabolic dysfunctions at an early stage in human pediatric populations, thus opening up new avenues in human clinical and behavioral nutrition research.

## Figures and Tables

**Table 1:** Ingredient composition of the high-fat diet (HFD).

Ingredient	g/kg of diet
Casein	258.46
L-cystine	3.88
Maltodextrin	161.54
Sucrose	88.91
Cellulose	64.62
Soybean oil	32.31
Lard	316.62
Mineral mix	12.92
Dicalcium phosphate	16.80
Calcium carbonate	7.11
Potassium citrate	21.32
Vitamin mix	12.92
Choline bitartrate	2.58

**Table 2:** Average daily food and water intake over a 14-day period.

	Diurnal			Nocturnal		
	RCD	HFD	*P*	RCD	HFD	*P*
Food intake (g/day)	9.52 ± 3.24	5.97 ± 1.30	**0.012**	18.47 ± 4.11	13.55 ± 0.87	**0.011**
Carbohydrate intake (g/day)	5.26 ± 1.79	1.57 ± 0.34	**<0.001**	10.20 ± 2.27	3.56 ± 0.23	**<0.001**
Protein intake (g/day)	1.71 ± 0.58	1.56 ± 0.34	0.541	3.32 ± 0.74	3.55 ± 0.23	0.424
Fat intake (g/day)	0.43 ± 0.15	2.08 ± 0.46	**<0.001**	0.83 ± 0.19	4.73 ± 0.30	**<0.001**
Water intake (mL/day)	8.34 ± 3.45	6.14 ± 1.68	0.128	24.32 ± 3.97	20.03 ± 5.05	0.079

Means ± SD; bold indicates significant difference between the two groups at the *P* < 0.05 level.
